# Minimally invasive local therapies for liver cancer

**DOI:** 10.7497/j.issn.2095-3941.2014.04.001

**Published:** 2014-12

**Authors:** David Li, Josephine Kang, Benjamin J. Golas, Vincent W. Yeung, David C. Madoff

**Affiliations:** ^1^Department of Radiology, Division of Interventional Radiology, New York-Presbyterian Hospital/Weill Cornell Medical Center, New York, NY 10065, USA; ^2^Department of Medicine, NYU Langone Medical Center, New York, NY 10016, USA; ^3^Flushing Radiation Oncology Services, Flushing, New York, NY 11354, USA; ^4^Department of Surgery, Division of Surgical Oncology, New York-Presbyterian Hospital/Weill Cornell Medical Center New York, New York, NY 10065, USA

**Keywords:** Liver, hepatocellular carcinoma (HCC), metastasis

## Abstract

Primary and metastatic liver tumors are an increasing global health problem, with hepatocellular carcinoma (HCC) now being the third leading cause of cancer-related mortality worldwide. Systemic treatment options for HCC remain limited, with Sorafenib as the only prospectively validated agent shown to increase overall survival. Surgical resection and/or transplantation, locally ablative therapies and regional or locoregional therapies have filled the gap in liver tumor treatments, providing improved survival outcomes for both primary and metastatic tumors. Minimally invasive local therapies have an increasing role in the treatment of both primary and metastatic liver tumors. For patients with low volume disease, these therapies have now been established into consensus practice guidelines. This review highlights technical aspects and outcomes of commonly utilized, minimally invasive local therapies including laparoscopic liver resection (LLR), radiofrequency ablation (RFA), microwave ablation (MWA), high-intensity focused ultrasound (HIFU), irreversible electroporation (IRE), and stereotactic body radiation therapy (SBRT). In addition, the role of combination treatment strategies utilizing these minimally invasive techniques is reviewed.

## Introduction

Primary liver cancer, particularly hepatocellular carcinoma (HCC), continues to be a growing global health problem and has risen to become the third most common cause of cancer-related deaths worldwide, accounting for over >800,000 deaths/year^1^^,^^2^. HCC is the fastest growing cause of cancer-related mortality both in the United States and worldwide over the past several decades due to both its increased incidence and poor prognosis^3^^,^^4^. In addition, the liver remains a common site of metastases, particularly from colorectal carcinoma^5^. It is estimated that up to two-thirds of patients with colorectal liver metastases ultimately die from their disease due to liver involvement^6^^,^^7^. Hence, there is an ever increasing need to develop effective treatments for both primary and metastatic liver tumors.

An extensive range of therapeutic options have been developed for the treatment of both primary and metastatic liver tumors. This has been, in part, driven by the limited effectiveness and range of available systemic treatments, particularly for HCC. Limitations of the effectiveness of systemic therapy are thought, in part, to be due to the frequent coexistence of HCC with cirrhosis and its high molecular heterogeneity. A significant advance was made in systemic therapeutic options with the randomized prospective validation of sorafenib in providing survival benefit in patients with HCC^8^; however, more recent randomized prospective controlled trials evaluating the use of Everolimus (EVOLVE-1) and Brivanib (BRISK-PS) have failed to demonstrate improvement in overall survival as compared to current standards of care^9^^,^^10^. Hence, the role of systemic therapies remains limited. Surgical resection and/or transplantation, locally ablative therapies and regional or locoregional therapies have filled the gap in liver tumor treatments, providing improved patient survivals as validated by prospective trials for both primary and metastatic tumors.

Surgical resection has been shown to result in improved survival outcomes and thus is the mainstay of curative therapy whenever amenable for both primary and metastatic liver tumors^11^^,^^12^. For example, in the setting of curative hepatectomy for colorectal metastases (CRM), the overall 5-year survival rate of surgical groups has approached >50%, as compared to approximately 15% for medical groups undergoing systemic chemotherapy alone^13^^,^^14^. However, surgical resection has potential complications, particularly in diseased liver states such as cirrhosis or prolonged chemotherapy^15^^-^^18^. There is reduced functional reserve to compensate for the resected hepatic parenchyma and in these settings, local ablation serves as a potentially curative option with the ability to induce complete cytotoxicity to the targeted tumor.

In addition to the increased incidence of HCC, improved screening, follow-up and imaging algorithms have led to the earlier detection and diagnosis of low volume disease primary and metastatic liver tumors amenable for minimally invasive local techniques. These factors have fueled an ever increasing need for local treatment options for both primary and metastatic liver tumors. The improved imaging algorithms have occurred in conjunction with advances in ablative techniques towards targeting of lesions. For percutaneous ablations, a wide range of guidance techniques have been developed including using ultrasound, computed tomography, magnetic resonance, fusion of imaging modalities, and needle tracking for optimal applicator positioning. Improved guidance and lesion localization has improved upon the technical feasibility of all minimally invasive local treatment options, further solidifying their use in current practice.

A wide range of minimally invasive local treatment techniques such as laparoscopic liver resection (LLR) and ablation have been developed. This article will highlight their role in current treatment paradigms, their principles of action, their relative differences in implementation, and the evidence for their use.

## Minimally invasive local therapies in current treatment paradigms

**Figure 1:**
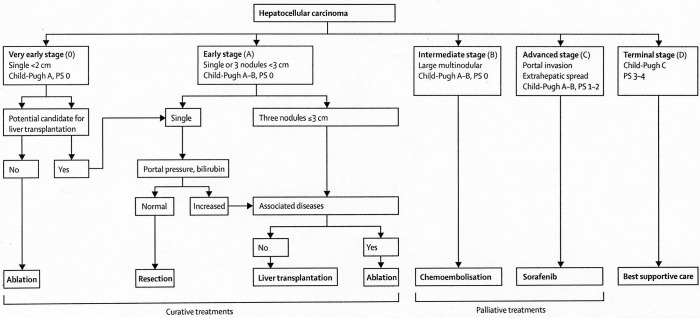
Barcelona clinic liver cancer treatment strategy. (Reprinted with permission from Forner *et al*.^2^).

The management of both primary tumors and metastases confined to the liver is best served with a multidisciplinary approach as adopted by multiple consensus guidelines, including the National Comprehensive Cancer Network (NCCN) and the European Association for the Study of the Liver (EASL)^11^^,^^12^^,^^19^. For HCC, multiple clinical staging systems have been developed in order to stratify patients towards appropriate treatments dependent on prognosis^20^. These staging systems typically stratify patients according to three generalized parameters: clinical performance status, extent of tumor, and liver function. One of the most widely adopted prognostic staging systems is the Barcelona Clinic Liver Cancer (BCLC) treatment strategy (**Figure 1**)^21^^,^^22^.

Volume of disease is an important determinant of prognosis and treatment options in patients with disease isolated to the liver. In the BCLC treatment strategy, tumor size and number directly alters disease staging, with a single tumor <2 cm considered very early stage (0), single or up to three tumors <3 cm considered early stage (A), or a large and/or multinodular tumor considered intermediate stage (B) in patients with equivalent Child-Pugh scores (A) and Performance Status (0). Potentially curative options such as surgical resection, liver transplantation (LT), or locally ablative therapies are reserved for patients with low volume disease (i.e., patients who are either BCLC stage 0 or A). For large or multinodular tumors with preserved liver function, locoregional therapies (i.e., transarterial embolotherapy) serve as the preferred treatment option.

Numerous locoregional treatment options have been explored for the treatment of large or multinodular tumors. Transarterially delivered therapies include bland embolization, chemoembolization, drug-eluting beads, and radioembolization, each with their relative advantages and drawbacks which are beyond the scope of this review^23^^-^^25^.

**Figure 2:**
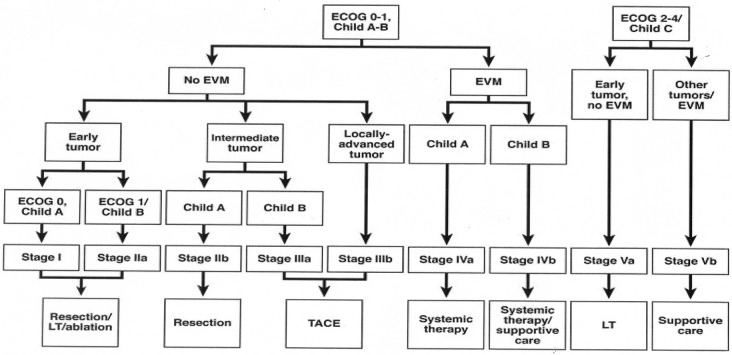
Hong Kong Liver Cancer prognostic classification and treatment strategy. (Reprinted with permission from Yau *et al*.^26^). Early tumor: ≤5 cm, ≤3 tumor nodules, and no intrahepatic venous invasion; Intermediate tumor: (1) ≤5 cm, either >3 tumor nodules or with intrahepatic venous invasion, or (2) >5 cm, ≤3 tumor nodules and no intrahepatic venous invasion; and Locally advanced tumor: (1) ≤5 cm, > 3 tumor nodules with intrahepatic venous invasion, or (2) >5 cm, >3 tumor nodules or/and with intrahepatic venous invasion, or (3) diffuse tumor. EVM, extrahepatic vascular invasion/metastasis; LT, liver transplantation.

Yau *et al*. recently analyzed a series of 3,856 patients from a single Asian center and created a new prognostic classification system and treatment strategy through statistical modelling methods termed the Hong Kong Liver Cancer (HKLC) schema (**Figure 2**)^26^. In their study, the HKLC was directly compared to the BCLC treatment strategy and was found to have increased ability to provide discriminable prognosis in terms of overall survival (area under receiver operating characteristic curve values, approximately 0.84 *vs*. 0.80; concordance index, 0.74 *vs*. 0.70). More importantly, HKLC identified subsets of BCLC intermediate and advanced stage patients who demonstrated a significantly increased survival benefit if classified under the HKLC system, where they would be stratified to receive more aggressive therapies such as surgical resection or local ablation. This new classification system further validates the effectiveness of surgical resection and local ablation in improving the overall survival in patients with primary liver cancer, and expands upon the eligibility criteria for patients to receive these more “aggressive” therapies.

Surgical resection has been shown to result in improved survival outcomes and thus is the mainstay of curative therapy whenever amenable for both primary and metastatic liver tumors^11^^,^^12^. However, surgical resection has potential complications, particularly in diseased liver states such as cirrhosis or prolonged chemotherapy^15^^-^^18^. In these settings, there is reduced functional reserve to compensate for the resected hepatic parenchyma. Even though portal vein embolization has been well-established to increase hepatic reserve in the anticipated future liver remnant, thus increasing the number of potential surgical candidates^27^; there still exists a large population of patients who are ineligible to safely undergo hepatic resection. In addition, comparison of surgical series to radiofrequency ablation (RFA) is limited by selection bias towards patient populations with greater comorbidities in the RFA cohorts^7^. Hence, locally ablative therapies maintain an important role in the treatment of liver tumors in patients with low volume of disease.

Traditional, open liver resection (OLR) is also affected by significant morbidity which directly impacts upon patient’s quality of life. As compared to surgical resection, local ablation is of relatively low cost, has minimal morbidity, and can be usually performed with shorter hospital stays^7^. Huang *et al*. prospectively evaluated 389 patients eligible for either surgical resection or percutaneous RFA for solitary HCC <3 cm in diameter for quality of life measures^28^. The authors found no difference in the two groups in disease-free and overall survival, however the RFA group demonstrated a statistically significant better health-related quality of life score (HRQL) as compared to the surgical group using the Functional Assessment of Cancer Therapy–Hepatobiliary (FACT-Hep) instrument. In their study, participants underwent open surgical technique with a right subcostal incision, with no comparison to laparoscopic approaches performed. As quality of life measures become increasingly important in decision of making of treatment paradigms, minimally invasive approaches such as laparoscopic resection and local ablation will continue to demonstrate increased clinical utility.

Techniques within the treatment armamentarium are not mutually exclusive and there is extensive reported evidence on the role of ablation in conjunction with surgical techniques.

For example, ablative techniques are commonly used to limit disease progression in patients awaiting transplantation (“bridge to transplantation”), with multiple studies demonstrating its utility in this setting^29^^-^^31^. RFA is most commonly used for bridge to transplantation, though there are several small reported series using microwave ablation (MWA) or stereotactic body radiation therapy (SBRT)^32^^,^^33^. Ablative techniques can be also performed in conjunction with major hepatectomy to offer curative options for select patients who have bilobar metastatic liver disease^13^^,^^34^. In addition, ablation can be performed in conjunction with locoregional therapies for improved therapeutic efficacy, as will be further elucidated below.

## Principles of action for local ablation

Multiple locally ablative techniques for the treatment of tumors have been developed and adopted into standard clinical practice. Each technique is based upon a different principle of action to induce cytotoxicity (**Table 1**).

**Table 1:** Ablative techniques and their mechanisms of action

Localized treatment modalities	Principle of action
Percutaneous ethanol injection (PEI)	Instillation of ethanol directly into the tumor causing cellular dehydration, protein denaturation, and occlusion of small vessels resulting in coagulation necrosis
Radiofrequency ablation (RFA)	Application of oscillating electrical currents resulting in resistive heating surrounding an electrode and tissue hyperthermia
Microwave ablation (MW)	Direct application of a propagating microwave energy level electromagnetic field to induce tissue hyperthermia via dielectric hysteresis
Irreversible electroporation (IRE)	Alteration of transmembrane potentials to induce irreversible disruption of cell membrane integrity
Percutaneous laser ablation (PLA)	Deposition of laser light via fiberoptic applicators to induce tissue hyperthermia
Cryoablation	Changes in gas pressures result in cooling of a cryoprobe in direct thermal contact with tumor resulting in ice crystal formation and osmotic shock
Stereotactic body radiation therapy (SBRT)	Extracorporeal coordinate system localization (+/- internal fiducial guidance) delivering multiple non-coplanar beams of photon beam radiation in a limited number of treatment fractions. This results in tumor cell death via direct DNA damage, as well as indirect DNA and cell damage through free radical formation
High-intensity focused ultrasound (HIFU)	Extracorporeal coordinate system focusing of multiple ultrasound beams into a focal point resulting in tissue hyperthermia and mechanical cavitation

The locally ablative techniques that rely on tissue heating for cytotoxicity result in a common pathway towards acute coagulative necrosis. Temperatures greater than 60 °C result in protein denaturation and near-instantaneous cell death. Temperatures in the range of 42-60 °C result in irreversible cell damage due to microvascular thrombosis, ischemia, and hypoxia^35^. Conversely, cryoablation results in cell death at temperatures less than –40 °C through both cell membrane disruptions by ice crystal formation, and rapid fluid shifts from osmotic gradients with associated cell membrane rupture. Irreversible electroporation (IRE) promotes cytotoxicity through irreversible damage to the cell membrane through the formation of permanent porous channels, stimulating apoptosis^35^. SBRT delivers high doses of photon beam radiation in a limited number of fractions (typically 3-6) to the target. The primary mechanism of cell death is through ionization of DNA, resulting in direct DNA damage and lethal double strand DNA breaks. Radiation also causes indirect DNA and cell damage through free radical formation^36^. Tumor cells are less efficient than normal cells in repairing radiation-induced damage, resulting in preferential killing of malignant cells^37^.

## Technical considerations and outcomes

### Laparoscopic liver resection (LLR)

While minimally invasive approaches have been widely adopted in many areas of surgery, there was initial apprehension regarding LLR^38^. These concerns were based on lack of familiarity with advanced laparoscopic techniques and equipment, challenges replicating open techniques, fears regarding bleeding and gas embolism, the possibility of port site metastases and peritoneal dissemination, unknown long-term outcomes, and lack of data from randomized trials.

**Figure 3:**
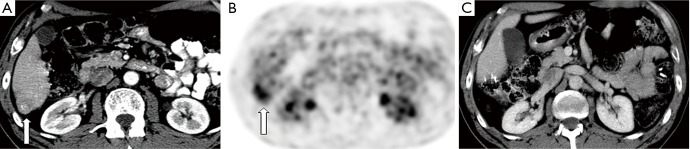
A 64-year-old female s/p right hemicolectomy for adenocarcinoma presenting with solitary segment 6 metastases discovered 3 years post-operatively on routine surveillance imaging. (A,B) Axial contrast enhanced CT image and corresponding PET image demonstrating FDG-avid segment 6 liver metastasis (arrow); (C) post-operative axial CT image obtained 8 years after laparoscopic liver resection demonstrating no residual or recurrent disease.

Despite this initial trepidation, over 3,000 LLR cases have been performed worldwide^38^. Most early LLRs were non-anatomic resections for peripherally located, benign lesions. Now, upwards of 50% of LLRs are performed for malignancy, with lobectomies and extended resections being undertaken in specialized centers (**Figure 3**). To date, no prospective randomized controlled trials have been conducted that compare LLR with OLR. Therefore, all existing comparisons are based on retrospective studies as well as meta-analyses. Taken together, published data purport the clinical benefits of LLR, without any oncologic compromise, particularly in the treatment of colorectal cancer (CRC) liver metastases.

Nguyen *et al*. performed a comparative analysis examining the benefits of LLR versus OLR in 31 case-cohort matched studies, which encompassed 2,473 patients^39^. For LLR versus OLR, there was significantly less blood loss (15 studies), less transfusions (4 studies; 0% LLR *vs*. 17.3% OLR; *P*=0.04), fewer postoperative days of narcotic medication (8 studies; 1 day LLR *vs*. 5 days OLR; *P*=0.001), quicker resumption of diet (7 studies; 1-2.4 days LLR *vs*. 2-4.3 days OLR; *P*=0.001 to <0.01), and decreased time to ambulation (1 study; 2.8 days LLR *vs*. 3.8 days OLR; *P*<0.005). While length of stay (LOS) information for liver surgery is variable secondary to cultural and institutional biases, almost all of the studies reported significantly reduced LOS after LLR (50% shorter) compared with OLR. Shorter LOS translated to reduced costs for LLR. The majority of studies reported comparable morbidity rates, while seven showed significantly lower complication rates with LLR (6%-13.8% LLR *vs*. 28.9%-47.8% OLR; *P*=0.001-0.04). There were no differences in operative time or mortality. While this review harbors the limitations inherent to any retrospective analysis of selected patients, it supports LLR as safe, feasible, and cost-effective with demonstrable benefits.

Of the nearly 3,000 published reports of LLR, approximately 50% were performed for malignant lesions, 35% of which were CRC metastases^38^. Multiple retrospective series have reported the safety, feasibility, and oncologic integrity of LLR for CRC^38^. Castaing *et al*. reported the only matched prospective comparison in patients undergoing resection of CRC liver metastases via laparoscopic (*n*=60) and open (*n*=60) approaches^40^. LLR was comparable to, and in some cases superior to, OLR in terms of oncologic outcomes. Specifically, the margin-free resection rate was greater in LLR *vs*. OLR (87% *vs*. 72%, *P*=0.04), while there was no significant difference in overall and disease-free survival. The 1-, 3-, and 5-year overall survival was 97%, 82%, and 64%, respectively, with LLR, versus 97%, 70%, and 56% with OLR (*P*=0.32). Furthermore, 1-, 3-, and 5-year disease-free survival was 70%, 47%, and 35%, respectively with LLR, versus 70%, 40%, and 27% in OLR (*P*=0.32).

The nearly 3,000 reported cases of LLR indicate that it is safe, feasible, and cost-effective with demonstrable short-terms benefits and no negative impact on long-term outcomes. LLR is a reasonable first-line approach in the treatment of CRC liver metastasis in select patients. LLR should only be performed in specialized centers by surgeons intimately acquainted with open and minimally invasive techniques. While a randomized prospective trial would be optimal to validate these results, no such study has been reported to date.

### Radiofrequency ablation (RFA)

The greatest accumulated evidence exists for RFA as compared to the other locally ablative techniques^41^. In RFA, electrical current is applied via an electrode(s) to tumors resulting in resistive heating and tissue hyperthermia. Tissues nearest to the electrode are heated most effectively, with more peripheral areas heated through thermal conduction^42^. As such, the mechanism of cytotoxicity in RFA is dependent on tissue impedance, with power deposition hindered in regions of high tissue impedance such as in surrounding lung or tissue that has undergone water vaporization (i.e., char tissue) immediately adjacent to the electrode due to rapid heating^41^^,^^43^^,^^44^. Multiple engineering designs have been developed to overcome the limitations caused by tissue impedance including multi-tined electrodes to expand the contact surface area, saline injection, and internal cooling. In addition, RFA requires the placement of grounding pads on the patient in order to close the electrical circuit, and skin burns related to the pads have been reported^45^^,^^46^. Fortunately, in current practice, skin burns are extremely rare since the routine use of larger grounding pads to allow for improved dispersion of thermal energy^47^. RFA is also limited by the “heat sink” effect where heat dissipation as a result of blood flow occurs, limiting its efficacy, with the effect more pronounced for lesions near the liver hilum^48^. For lesions in this location, at thermal energies necessary to obtain adequate thermal ablative margins, RFA is limited by bile duct injury ultimately resulting in stricture^47^.

**Figure 4:**
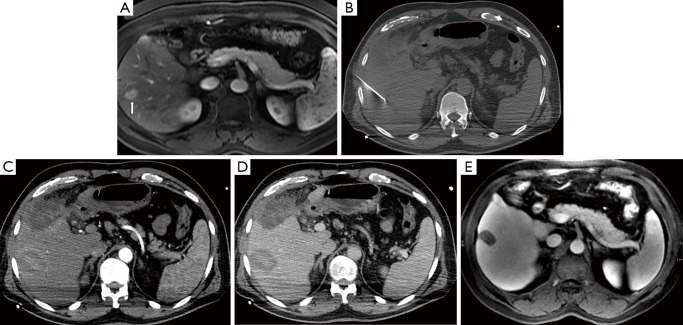
A 56-year-old male with hepatitis C complicated by hepatocellular carcinoma, not a surgical candidate, presenting with an isolated tumor in segment 6 of the liver. (A) T2 weighted axial MRI image demonstrates lesion in segment 6 (arrow). (B) Non-contrast axial CT image during procedure with applicator in the hypoattenuating mass. Immediate contrast-enhanced post-RFA axial CT images in arterial (C) and portal venous (D) phases show complete ablation in the area of the tumor. (E) T2 weighted axial MRI image at two year follow-up demonstrates complete necrosis of the segment 6 tumor. RFA, radiofrequency ablation.

RFA has been used extensively in the setting of both primary and metastatic liver tumors (**Figure 4**). Weis *et al.* recently published a Cochrane database analysis on the use of RFA for the treatment of HCC^49^. The authors identified and included 11 randomized clinical trials with a total of 1,819 participants with four comparisons: RFA *vs*. hepatic resection (three trials, 578 participants^50^^-^^52^); RFA *vs*. PEI (six trials, 1,088 participants^53^^-^^58^); RFA *vs*. MWA (one trial, 72 participants^59^), and RFA versus percutaneous laser ablation (PLA) (one trial, 81 participants^60^) with the primary outcome measure being overall survival. After analysis, the authors concluded that there was moderate quality of evidence that hepatic resection is superior to RFA regarding survival; however, RFA might be associated with fewer complications and shorter hospital stay. They also found moderate quality evidence that RFA is superior to PEI in regards to survival. There was insufficient evidence to make firm conclusions regarding RFA, in comparison to locally ablative techniques such as MWA or PLA. Similar conclusions can be drawn regarding the clinical utility of RFA for treatment of metastatic lesions to the liver. A summary of studies comparing RFA versus surgery for liver metastases published since 2007 is shown in **Table 2**^61^^-^^72^. Surgical resection was found to be superior to RFA in overall survival when feasible.

**Table 2:** Surgery versus RFA

Study and year	Type of study	Treatment	No. of patients	Tumor size (range) (cm)	Overall survival (%)			*P*	Disease free survival (%)		*P*
					2 years	3 years	5 years		3 years	5 years	
White *et al*., 2007^61^	R	Surg	30	2.5 (1.0-5.0)	100	82	65	N/A	51	36	N/A
		RFA	22	2.0 (1.0-5.0)	100	28	0		0	0	
Gleisner *et al*., 2008^62^	R	Surg	192	3.5 (2.0-5.0)		72	54	0.01	41	41	0.01
		RFA	11	2.5 (1.9-4.0)		51	28		9	0	
Berber *et al*., 2008^63^	R	Surg	90	3.8		70	40	0.35	45	38	N/A
		RFA	68	3.7		35	30		29	0	
Lee *et al*., 2008^64^	R	Surg	116	3.3 (0.5-18.0)		51	66	0.23	88	85	N/A
		RFA	37	2.25 (0.8-5.0)		32	49		53	43	
Hur *et al*., 2009^65^	R	Surg	42	2.6 (0.6-8.0)		70	50	0.026			0.03
		RFA	25	2.5 (2.8-3.6)		60	26				
Reuter *et al*., 2009^66^	R	Surg	192	5.3		55	23	NS	42	24	N/A
		RFA	66	3.2		42	21		24	8	
McKay *et al*., 2009^67^	R	Surg	58	4.1 (1.5-14.5)		60	43	0.021			
		RFA	43	3.0 (1.0-7.5)		39	23				
Otto *et al*., 2010^68^	P	Surg	28	5.0 (1.0-15.0)		60	51	0.721	40	30	N/A
		RFA	82	2.0 (1.0-5.0)		67	48		18	18	
Schiffman *et al*., 2010^69^	R	Surg	92	5.6	92	81	65	0.005			
		RFA	81	3.9	81	64	42				
Lee *et al*., 2012^70^	R	Surg	25	4.0 (0.7-9.7)		68	44	0.001	40	12	0.004
		RFA	28	2.0 (1.0-4.8)		36	18		11	0	
Kim *et al*., 2011^71^	R	Surg	278	2.6		59	45	0.007	32	28	0.004
		RFA	177	2.1		50	36		26	20	

P, prospective; R, retrospective; RFA, radiofrequency ablation.

However, understanding the role of RFA in comparison to surgical resection has been confounded by both inhomogeneity in both treatment techniques and patient populations between the two groups. Two prospective randomized studies for RFA versus surgical resection were determined by Weis *et al*.^49^ to have the smallest degree of potential bias: 168 patients collated by Feng *et al*.^52^ and 230 patients by Huang *et al*.^50^. In both studies, the authors achieved RFA zones with greater than 0.5 cm margins on the initial hospitalization prior to discharge, and open surgical resection was performed with the assistance of intraoperative ultrasound to ensure complete tumor resection. Though these studies demonstrated improved overall local recurrence rates with resection as compared to RFA, it came at the cost of increased morbidity with increased hospital stays and adverse events. A recent study by Lee *et al*. demonstrated that the cohort who underwent surgical resection was younger, with better liver function reserve and performance status than those who underwent RFA^73^. When accounting for this discrepancy in populations using propensity score analysis, RFA was found to be superior to surgical resection for patients with small HCC and Child-Pugh Turcotte scores of 5.

### Microwave ablation (MWA)

In MWA, local tissue hyperthermia is created through the direct application of an electromagnetic field which causes dielectric hysteresis^35^. As such, MWA can readily penetrate through various tissue types including those with high impedance such as lung or char tissue, where RFA is limited^35^^,^^41^. High tissue temperatures can be achieved with MWA, allowing for increased efficacy of ablations as compared to RFA. In addition, pre-clinical data suggests MWA is not as influenced by “heat-sink” effects next to major vessels as compared to RFA, where heat dissipation occurs as a result of blood flow^74^. Though grounding pads are not necessary for MWA, burns still remain as a potential complication since high temperatures can be achieved which propagate along the microwave applicator shaft that result in entry site injury^41^.

**Figure 5:**
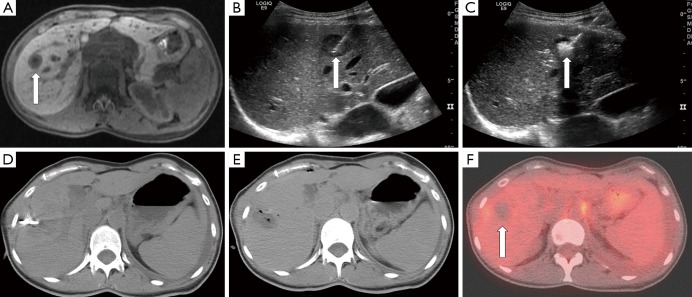
A 40-year-old female with metastatic breast carcinoma who presents with a focal metastatic tumor to segment 7 of the liver. (A) Axial post-contrast MRI image demonstrates 1.7×1.1 cm^2^ hypointense mass in segment 7 (arrow). Intraprocedural ultrasound images (B) During needle placement (arrow), (C) During ablation. Note the hyperechoic area which represents microbubble formation during heating (arrow). (D) Non-contrast axial CT image during procedure demonstrates applicator in place. (E) Non-contrast axial CT image immediately after ablation shows a hypodense region with focal air bubbles indicative of the ablation zone. (F) Fused axial PET-CT image 3 months post-ablation demonstrating ablation cavity with no evidence of residual FDG-avidity (arrow).

Given its increased efficacy of ablation and shorter time to achieve ablations, MWA has increasingly been used in the treatment of both primary and metastatic tumors of the liver (**Figure 5**). Ding *et al*. recently evaluated a series of 198 patients (85 RFA/113 MWA) with HCC meeting Milan criteria and found similar disease-free survival, cumulative survival, and complication rates between the two groups^75^^,^^76^. In their series, all patients were BCLC Stage A, and tumor size was equivalent between the two groups (mean tumor diameter 2.38±0.81 cm RFA cohort; 2.55±0.89 MWA cohort). Shibata *et al*. compared the efficacy of MWA versus RFA in a series of 72 patients (36 RFA/36 MWA) in a randomized fashion from a cohort of patients with equivalent background demographics and mean tumor size and concluded that therapeutic effects, complication rates, and rates of residual untreated disease were equivalent between the two modalities^59^. Zhang *et al*. evaluated overall survival, complete ablation, local tumor progression and distant recurrence in a series of 155 patients (mean tumor size 2.3±0.4 cm RFA cohort and 2.2±0.4 cm MWA cohort) and also found that RFA and MWA were equivalent^77^.

### External beam radiation therapy including SBRT

Historically, the use of external beam radiation therapy in treatment of liver tumors has been limited due to the overall low tolerance of liver tissue to radiation^78^. Radiation produces tumor cell kill by depositing energy within atoms, causing transformation into free radicals. This results in direct DNA damage, as well as indirect DNA and cellular damage through generation of reactive oxygen species. Ultimately, generation of DNA double strand breaks leads to tumor cell death. Radiation can achieve excellent tumor control when delivered to ablative doses^79^, but dose is limited due to the radiation tolerance of the surrounding normal liver tissue and adjacent organs. Radiation-induced liver disease is a feared complication of treatment, classically manifesting as a triad of anicteric hepatomegaly, ascites, and elevation of alkaline phosphatase.

With recent advances in technology, radiation can be directed to the tumor while minimizing exposure of surrounding normal liver. Imaging techniques have improved, allowing for precise delineation of hepatic tumors. Breathing motion control and image guidance both before and during treatment delivery permit tumor-directed treatment with accurate localization^80^^,^^81^, reducing treatment uncertainty and decreasing the margin of error. Treatment planning techniques and machines has also improved, allowing highly conformal treatment delivery. With increased conformality comes the potential to deliver higher doses of radiation and thereby increase local control without increasing toxicity. As a result of these advances, radiation is being re-explored as a treatment modality for both primary and metastatic liver tumors.

Over the past decade, multiple studies have been published on the use of conformal radiation treatment for hepatic malignancies, and results have been favorable with high rates of local control^79^^,^^82^^-^^88^. Though majority of these studies were small, and many are retrospective, they have provided ample background data to establish current prospective studies and randomized trials. Currently open to accrual is Radiation Therapy Oncology Group (RTOG) 1112, a phase III protocol randomizing patients with unresectable HCC to monotherapy with sorafenib, or sequential tumor-directed radiation followed by sorafenib^89^.

**Figure 6:**
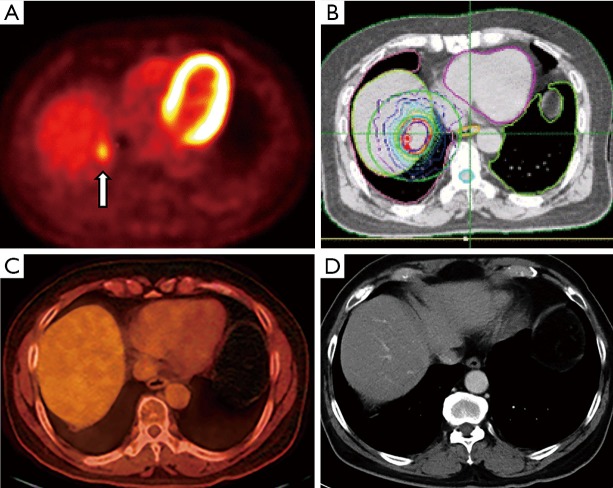
A 61-year-old male with history of localized rectal cancer treated with chemoradiation and surgery; subsequently diagnosed 2 years later with isolated liver lesion growing in size and avidity, biopsied positive for metastatic rectal cancer. Patient initially refused all invasive procedures including surgery, opting for chemotherapy alone, then ultimately agreed to SBRT. (A) Pretreatment axial fused PET-CT image demonstrating metastatic segment 7 liver lesion with SUV 3.8 (arrow). (B) Treatment plan depicting prescription isodose line (in red) with rapid dose fall off around the tumor. (C) Axial fused PET-CT obtained 18 months after SBRT demonstrating no evidence of disease. (D) Axial contrast enhanced CT image obtained 4 years after SBRT demonstrating no evidence of disease. SBRT, stereotactic body radiation therapy.

SBRT is a recently developed technique that allows high conformal radiation treatment by utilizing multiple, non-coplanar beams or arcs to target the tumor with millimeter precision. Compared to standard external beam radiation (3-D or intensity modulated RT), SBRT can create a rapid radiation dose fall off, allowing ablative radiation doses to be delivered to gross tumors while sparing adjacent tissue (**Figure 6**). As a result, SBRT has emerged as the primary technique of delivering radiation to liver tumors.

SBRT treatment is delivered as follows. First, to reduce uncertainty from breathing motion, internal fiducial marker placement is utilized in many institutions to allow tumor tracking. Additional ways to account for or restrict liver movement include use of breath hold technique, abdominal compression or respiratory gating. Internal placement of fiducial markers is often performed to facilitate tumor targeting and tracking. Typically, gold fiducials are used and placed in vicinity of the tumor approximately 1 week before treatment planning CT, and typically at least two or more fiducials are placed in non-co-planar fashion. Markers are placed percutaneously with image guidance under local anesthesia; it is an outpatient procedure with standard risks from introducing a needle into the liver (bleeding, infection, seeding, pain) and small risk of fiducial migration^90^. After fiducial placement, a pre-treatment CT is obtained for radiation planning purposes; this is ideally performed with multi-phasic IV contrast in exhale breath-hold position. A diagnostic MRI or CT is also utilized to define the tumor volume. The gross tumor volume (GTV) is contoured by the radiation oncologist on each slice of the pre-treatment CT. A clinical target volume (CTV) can be added to account for microscopic extension; in many cases, there will be no CTV expansion necessary. Finally, a planning target volume (PTV) expansion is added to the GTV to account for daily setup error and internal organ motion. The size and number of lesions that can be targeted, and dose of radiation that can be delivered, is dependent primarily on normal liver reserve and estimated risk of liver complications. Patients with poor liver function may require dose reduction to reduce the likelihood of complication^85^. Childs Pugh class is one measure of estimating normal liver function; for Childs Pugh category B, reduction in radiation dose may be a consideration. Childs Pugh category C is less commonly treated to ablative doses, given poor functional reserve and high risk of toxicity. An alternate method of estimating normal liver function is measurement of Veff, and is currently utilized in RTOG 1112^89^. Veff is utilized as an aid in dose prescription, along with standard metrics such as the mean liver dose. For example, for a five fraction treatment, the prescribed total dose ranges from 27.5 to 50 Gy depending on the effective liver volume^89^. At least 700 cc of normal liver should receive less than 15 Gy in order to maintain a <5% risk of RILD^78^.

Typically, treatment is delivered in 3-6 fractions, with minimum 1-3 days between each fraction. Depending on location, it is possible to target multiple tumors in a single fraction. The actual radiation treatment is less than 1 hour in duration. Because it is non-invasive and painless, no sedation or anesthesia is required.

The utility of SBRT as a treatment for unresectable liver tumors was first reported in 1995 by Blomgren and colleagues^91^. Since then, there have since then been several additional series reporting excellent local control outcomes demonstrating low toxicity, feasibility and efficacy^83^^-^^85^^,^^87^^,^^91^^,^^92^. In general, treatment is delivered in 3-6 fractions, to total doses ranging from 30-60 Gy. With the more generally adopted dosing regimens, overall local control rates for small liver tumors (6 cm or less) ranges from 70%-90% at 2 years. It has also been shown that higher doses are associated with improved local control^82^. Toxicity rates are associated with poor baseline liver dysfunction, stressing the importance of careful patient and dose selection^85^. **Table 3** provides a summary of recent phase I/II studies that have reported treatment outcomes for primary and/or metastatic liver tumors^79^^,^^82^^,^^83^^,^^85^^,^^86^^,^^88^^,^^93^^,^^95^^,^^96^.

**Table 3:** Phase I/II studies on liver SBRT

Study and Year	Type of Study	Histology	No. of patients (No. of tumors)	Tumor size (range) (mL)	Dose	Median FU (m)	Child-Pugh class	Local control	Overall survival	≥ G3 acute toxicity (%)
Méndez Romero *et al*., 2006^85^	Phase I/II	met (*n*=34), HCC (*n*=11)	25 (45)	22.2 (1.1-322)	10-12.5 Gy × 3^ǂ^	12.9	Child-Pugh A (*n*=5), Child-Pugh B (*n*=2); rest N/A	1y 94%; 2y 82%	1y 75%; 2y 40%	G3 (12%): 2 liver enzyme elevation; 1 asthenia; G5 (4%): liver failure
Tse *et al*., 2008^88^	Phase I	HCC, IHC	41	173 (9-1,913)	24-54 Gy in 3 fx	17.6	Child-Pugh A	1y 65%	Median 13.4 m	G3 (12%): 6 liver enzyme elevation
Cárdenes *et al*., 2010^83^	Phase I	HCC	17 (25)	34 (8-95)	Dose esc to 48 Gy in 3 fx	24	Child-Pugh A (*n*=6), Child-Pugh B (*n*=11)	1y 100%	1y 75%; 2y 60%	Child-Pugh A: no DLT. Child-Pugh B: ≥G3 hepatic toxicity
Rusthoven *et al*., 2009^79^	Phase I/II	met (31.9% CRM)	47 (63)	14.9 (0.8-98.0)	Dose esc to 60 Gy in 3 fx	16		1y 95%; 2y 92%	Median 20.5 m	G3 (2%): 1 soft tissue toxicity
Lee *et al*., 2009^93^	Phase I	met (58.8% CRM)	68 (143)	75.2 (1.2-3,090)	60 Gy in 6 fx^ǂ^	10.8	Child-Pugh A	1y 71%	Median 17.6 m	G3 (6%): 2 gastritis, 2 nausea, 1 lethargy, 1 thrombocytopenia; G4 (1%): 1 thrombocytopenia
Goodman *et al*., 2010^94^	Phase I	met (*n*=10), HCC (*n*=2), IHC (*n*=5)	26 (40)	32.6 (0.8-146.6)	Dose esc to 18 Gy in 1 fx	17.3	Child-Pugh A	1y 77%	Median 28.6 m	No acute G3 or higher acute toxicity
Kang *et al*., 2012^95^	Phase II	HCC	50 (56)	14.9 (2.4-213.8)	42-60 Gy in 3 fx	17	Child-Pugh A (*n*=41), Child-Pugh B (*n*=6)	2y 94.6%	2y 68.7%	G3 (6%): 3 GI ulcers; G4 (4%): 2 gastric ulcer perforation
Scorsetti *et al*., 2013^96^	Phase II	met (47.5% CRM)	61 (76)	54.9 (1.8-134.3)	75 Gy in 3 fx^ǂ^	12		1y 94%	Median 19 m	G3 (1.7%): 1 chronic chest wall pain
Bujold *et al*., 2013^82^	Sequential phase I/II	HCC	102	117 (1.3-1,913)	24-54 Gy in 6 fx	31.4	Child-Pugh A	1y 83%	Median 17 m	G3 (27%): increased AST/ALT most common G4 (3%): 2 bilirubin increase ; 1 liver failure; G5 (7%): 5 liver failure, 1 GI bleed, 1 cholangitis

^ǂ^, dose reduction if necessary for poor baseline liver function/to maintain constraints for organs at risk. SBRT, stereotactic body radiation therapy; CRM, colorectal metastases; CTV, clinical target volume; esc, escalation; DLT, dose limiting toxicity; met, metastatic liver tumors; fx, fractions; G3, grade 3; IHC, intrahepatic cholangiocarcinoma; m, months; n, number;  N/A, not available.

The single institution phase I/II trial by Mendez Romero *et al*.^85^ was an initial study with promising results, that provided rationale for subsequent investigation. In this report, 34 metastatic and 11 primary liver tumors with treated with SBRT. Median tumor size was 3.2 (range, 0.5-7.2) cm. Dose prescribed ranged from 10-12.5 Gy × 3 fractions, or 5 Gy × 5. Local control rates were 94% and 84% at 1 and 2 years, respectively. Four patients had grade 3 or higher acute toxicity, including a grade 5 toxicity in a Child-Pugh class B HCC patient, who developed liver failure. A phase I trial by Goodman *et al*. recently explored the use of single fraction SBRT for primary and metastatic liver tumors, demonstrating comparable efficacy with low toxicity^94^. However, single fraction treatment has not been widely adopted into clinical practice outside the confines of a clinical trial. The current ongoing RTOG 1112 trial delivers SBRT to unresectable HCC in 5 fractions of 5.5 to 10 Gy, depending on liver reserve, or effective liver volume irradiated (Veff).

The impact of SBRT on overall survival is yet to be determined. The hetereogeneity of baseline liver function, variation in tumor size and comorbidities in the patient population of reported series precludes an accurate assessment of overall survival and local control outcomes across varying treatment modalities. This will need to be addressed in the future through randomized clinical trials. Preliminary studies suggest that liver SBRT is a non-invasive and effective treatment modality for liver tumors in non-surgical candidates who are ineligible, or have failed other treatment modalities.

### High dose rate (HDR) CT guided brachytherapy

Radiation has a dose-dependent effect on local control, but deliverable dose is limited by surrounding critical structures. Another technique of radiation delivery that has shown promising outcomes in single-institution series is HDR CT-guided interstitial brachytherapy^97^^-^^99^. Radiation is delivered using an iridium-192 source as a single fraction. The benefit of this technique is that radiation dose fall-off from the iridium-192 source is quite dramatic, allowing greater protection of the surrounding healthy liver compared to external radiation techniques. A prospective phase II trial by Ricke *et al*.^100^ demonstrated favorable results with this technique for patients with liver tumors near the hilum, or large (>5 cm) tumors. With average dose of 17 Gy, local control at 12 months was 71% and 40%, respectively. Subsequent series have shown promising local control for large tumors. Collettini *et al*. reported outcomes of 35 patients with HCC, ranging in size from 5-12 cm, treated with HDR brachytherapy. All patients were Childs Pugh A or B. At 12 months, local control was 93% and no major toxicity was reported^101^. In conclusion, HDR brachytherapy may be another alternative for liver tumors that are not amenable to traditional ablative techniques, and further studies with longer follow up are warranted.

### High-intensity focused ultrasound (HIFU)

HIFU incorporates multiple ultrasound beams produced by piezoelectric or piezoceramic transducers directed into a three-dimensional focal point typically 1 to 5 mm in diameter and 10 to 50 mm in length^102^. The ultrasound beams delivered to the focal point are both thermally ablative and causes cavitation to the underlying tissues. Coupling of the ultrasound source and the patient is achieved through a degassed water bath to achieve minimal reflection or absorption of the sound waves prior to reaching the focal point. Motion needs to be minimized during the entire procedure and the focal zone is shifted sequentially to cover an entire area of interest for ablation. Hence, patients are typically in a confined space for at least several hours for the procedure, and general anesthesia is recommended for patient comfort.

Several series have evaluated the safety and efficacy of HIFU for the treatment of HCC^103^^-^^109^. Ng *et al*. reported on the safety and efficacy of HIFU on the treatment of HCC (median tumor size 2.2 cm; range, 0.9 to 8 cm) for a series of 49 patients who were not surgical candidates and concluded that HIFU was an effective modality in this setting, with 1- and 3-year overall survival rates reported as 87.7% and 62.4%, respectively^106^. Wu *et al*. reported on the safety and efficacy of large HCCs (mean tumor diameter 8.1 cm; range, 4 to 14 cm) treated with HIFU and found favorable overall survival rates of 86.1%, 61.5%, and 35.3% at 6, 12, and 18 months, respectively^107^. Recently, Cheung *et al.* reported on the outcomes of HIFU performed for the treatment of HCC as a bridge to transplantation in 10 patients as compared to 29 patients who received transarterial chemoembolization and found excellent efficacy with HIFU (90% complete response/10% partial response) with none of the patients on the liver transplant list (*n*=5) dropping out^103^. Studies investigating the use of HIFU for the treatment of metastatic liver tumors are limited. Most studies do not address outcomes related to this defined subset of patients. Wu *et al.* reported on the largest series of patients who have undergone HIFU (*n*=1,038); however histologies and tumor locations were variable^108^. In their study, primary and metastatic liver tumors were reported on as a single cohort without analysis of the subgroups. Overall, these studies indicate that HIFU may serve as an excellent locally ablative technique for the treatment of HCC; however, technical implementation of the procedure serves as a significant barrier to widespread use.

### Irreversible electroporation (IRE)

IRE is an ostensibly non-thermal technique in which the direct placement of electrodes is used to create a pulsed direct current inducing cytotoxicity in tumor cells by altering transmembrane potentials which irreversibly disrupt cell membrane integrity^110^. As compared to other percutaneous approach techniques, IRE requires the placement of at least two applicators in parallel to create ablation zones in the range of 1.5-2.0 cm per electrode pair^111^. The zone of ablation created by IRE is dependent on multiple factors including electrode spacing, relative positions, active tip length, pulse number, pulse duration, and applied voltage^110^^,^^112^. Hence, precise placement of at least two probes is necessary to create appropriate zone of ablations, making IRE more technically challenging than other locally ablative techniques. The current generated by IRE is known to cause whole-body muscle contractions and general anesthesia with the use of neuromuscular blockage is obligatory for its clinical use. In addition, IRE has been shown to induce cardiac arrhythmias, though this potential complication can be averted with the use of cardiac synchronization of the administered pulses to the complete refractory period of the cardiac cycle^113^.

IRE has a theoretical safety advantage over other locally ablative techniques in the treatment of tumors close to structures susceptible to thermal injury, such as major bile ducts in the liver. In addition, IRE may be more effective in cytotoxicity for tumors next to major vessels, due to reduction in the heat-sink effect. Several small series have reported on the use of IRE for the treatment of liver tumors in locations thought to be unfavorable for other ablative therapies^112^^,^^114^^-^^117^. In a series of 18 HCC lesions in 11 patients treated with IRE by Cheung *et al.*, 13/18 (72%) of lesions were completely ablated [13/14 (93%) of lesions <3 cm] with a local recurrence-free period of 18±4 months^115^. Cannon *et al*. reported on 44 patients with both primary (*n*=14) and metastatic (*n*=20 colorectal; *n*=10 other) liver tumors treated with IRE; with local recurrence free survival of 97.4%, 94.6%, and 59.7% at 3, 6, and 12 months, respectively^114^. In their series, a trend towards higher recurrence rates for tumors >4 cm was identified (HR, 3.236; 95% CI, 0.585-17.891; *P*=0.178). Kingham *et al*. reported on the use of IRE in a series of 28 patients with 65 tumors (median 1 cm) and found a local recurrence rate of 5.7% at median follow-up of 6 months^116^. These small series suggest that IRE has improved efficacy in smaller lesions; and though local tumor control is excellent at 3-6 months, recurrence rates are higher after 12-18 months.

### Percutaneous ethanol injection (PEI)

PEI involves the direct instillation of ethanol into tumors ultimately resulting in coagulation necrosis. As such, the technique is relatively simple and inexpensive. In practice, PEI is limited by poor and uneven distribution of the ethanol within the tumor and diffusion into the adjacent normal tissues. Ultrasound guidance allows for real-time monitoring of the ethanol dispersion and is generally used to help compensate for these limitations. Even so, multiple intra-procedural needle repositions are sometimes needed, given the uneven distribution of the ethanol^118^. The longest clinical experience and follow-up for patients is available for PEI in the treatment of HCC, with some studies documenting observation periods greater than 15 years^119^^,^^120^. However, as discussed in the RFA section, multiple studies have demonstrated superiority of RFA to PEI in patient overall survival. Hence, other locally ablative therapies have been favored in clinical practice as compared to PEI.

### Cryoablation

Cryoablation involves the direct application of a cryoprobe into a tumor. The thermal contact with the tumor results in ice crystal formation and osmotic shock. One distinct advantage of cryoablation as compared to other ablative techniques is that the zone of ablation is readily visible (i.e., “iceball”) using non-contrast CT, ultrasound, or MRI monitoring, allowing for precise targeting of a zone of ablation to the tumor^121^. In addition, multiple probes can be used in tandem to create larger ablation zones and shorten procedural times. Despite the technical advantages of cryoablation, its complication profile has limited its general use in the treatment of liver tumors. Though uncommon, cryo-shock is a potentially life-threatening complication distinct to cryoablation, characterized by thrombocytopenia, acute renal failure, adult respiratory distress syndrome and disseminated intravascular coagulopathy^121^. A meta-analysis performed by Huang *et al.* investigated the role of cryoablation in comparison to RFA in the treatment of unresectable HCC^122^. Outcomes analyzed included mortality, complication rate and local recurrence; and RFA was found to be superior to cryoablation, particularly in regards to complication rates (OR 2.80; 95% CI, 1.54-5.09) and local tumor recurrence (OR 1.96; 95% CI, 1.12-3.42).

### Percutaneous laser ablation (PLA)

PLA involves the direct deposition of laser light via fiberoptic applicators to induce tissue hyperthermia in tumors. Proponents of PLA advocate that the thin flexible fiberoptic delivery fibers allow for safer and technically easier approaches to tumors as compared to other ablative techniques^123^. In addition, ablative zones can be controlled with feedback and dose-planning systems. Hence, low complication rates are associated with PLA. Vogl *et al*. reported on the use of PLA in 899 patients with 2,520 liver tumors and demonstrated a major complication rate of 2%; the majority being either pleural effusion (*n*=16; 0.8%) requiring thoracentesis or hepatic abscess requiring drainage (*n*=15; 0.7%)^124^. Evidence regarding the use of PLA for the treatment of liver tumors is limited. Pacella *et al*. retrospectively analyzed the use of PLA for the treatment of HCC in a cohort of 432 cirrhotic patients (344 with a single nodule ≤4 cm; 88 with two or three nodule <3 cm)^125^. An initial complete response was reported in 344 patients (78%) with median overall survival of 47 months (95% CI, 41 to 53 months). Multivariate analysis confirmed the achievement of complete ablation (*P*=0.001; RR =0.517) as an independent predictor of survival. Ferrari *et al.* evaluated the use of PLA as compared to RFA in a prospective randomized study of 81 cirrhotic patients with HCC^60^. There was a homogeneous distribution in age, sex, Child-Pugh class and HCC nodule dimensions between the two cohorts with RFA found to have better initial tumor ablation as compared to PLA (94% *vs*. 78%). In addition, it was suggestive that the RFA cohort had improved overall survival as compared to PLA at 1, 3 and 5 years (92.2% *vs*. 88.6%, 75.0% *vs*. 70.4%, 40.9% *vs*. 22.9%; respectively), however the difference did not reach statistical significance (*P*=0.3299). Though the evidence is still limited, there is suggestion that PLA is limited in achieving complete tumor ablation as compared to other locally ablative therapies.

## Combination treatment approaches

There are multiple theoretical advantages in combining locally ablative therapies with each other or to other treatment options such as transarterial embolotherapy. Several of the described modalities have different principles of action in creating cytotoxicity and combining them may potentiate their effectiveness, improving tumor control. In combination, dose and energy profiles for each locally ablative modality may be potentially reduced without comprising cytotoxicity to tumor cells; increasing the safety margin to adjacent normal tissues. Combination approaches also allow for limitations of each modality on its own to be overcome, thus expanding the number of patients eligible for therapeutic options.

The greatest cumulated evidence exists for the use of RFA in combination with transarterial embolotherapy. In theory, the decreased blood flow induced by transarterial embolization reduces heat loss, improving the RFA margins. In addition TACE enhances nearby control of satellite lesions^126^. Conceptually, the practice is similar to chemotherapy in addition to surgical resection for oncologic treatments. Ablation provides localized curative treatment of the tumor similar to surgery while TAE provides control of micrometastases similar to chemotherapy. In 2013, several meta-analyses cumulated the randomized controlled trials available for RFA plus TACE as compared to RFA alone^127^^-^^129^. All of the meta-analyses concluded that there was high quality evidence suggesting that TACE in combination with RFA improved survival outcomes as compared to RFA alone for patients with HCC, particularly for tumors larger than 3 cm in size. Peng *et al*.^130^ studied a series of 189 patients with HCC less than 7cm, randomly assigned to TACE plus RFA or RFA alone and found that the TACE plus RFA cohort had improved overall and recurrence free survival (HR, 0.525; 95% CI, 0.335-0.822; *P*=0.002; HR, 0.575; 95% CI, 0.374-0.897; *P*=0.009, respectively).

The use of localized radiation in combination with thermal ablation is of particular interest for future investigation. Hyperthermia has been proven to potentiate the cytotoxic effect of radiation and has been clinically adopted^131^^,^^132^. In addition, recent animal studies have suggested that the use of radiation in combination with RFA resulted in improved tumor growth control as compared to RFA alone^133^^,^^134^. In a review by Ahmed *et al*., the combination of therapies were found to have increased tumor necrosis, decreased tumor growth and improved overall animal survival in a rat tumor model^41^. The mechanism for this potentiation is still unknown. One potential etiology is from increased blood flow after ablation resulting in improved oxygenation which potentiates free radical formation with subsequent radiation. Another is increased free-radical formation after radiation resulting in inhibition of tumor cell recovery which potentiates the effects of thermal ablation^41^.

The combination of thermal ablation in tandem with SBRT is complimentary from a technical standpoint and is readily adaptable into clinical practice. Thermal ablative techniques offer the added clinical benefit of tissue diagnosis that can be obtained immediately prior to the procedure that is usually necessary for radiation oncologists to appropriately initiate treatments. In addition, fiducial seed placement sometimes necessary for SBRT localization can be performed at the same time. Conversely, SBRT can provide improved tumor control in areas where thermal ablation is known to have high failure rates, such as next to major vessels in the setting of RFA. In addition, doses for radiation and ablation zones for thermal ablation can be reduced to preserve normal tissue parenchyma without compromising efficacy in tumor control. The combination of these therapies serves as an exciting avenue of research with clinical studies necessary to validate proper dosing and timing regimens.

## Conclusion

Minimally invasive therapies for the treatment of both primary and metastatic liver tumors continue to highlight the fact that we are privileged to practice medicine during a time of such dynamic change and innovation. As such, both laparoscopic resection and locally ablative therapies have solidified a role in the treatment paradigms of both primary and metastatic liver tumors, including the most recently validated prognostic treatment strategy, the HKLC schema.

Based upon its principle of action and technical aspects of implementation, each local therapy has its benefits and drawbacks in clinical practice. The greatest cumulative evidence exists for surgical resection and RFA. As both cost and quality of life become further incorporated into treatment paradigms, minimally invasive local therapeutic options will continue their trends towards increased utilization.

As summarized in the principles of action section, locally ablative therapies harness the newest technological developments into various devices that are able to induce localized cytotoxicity to tumor cells while minimizing damage to nearby native tissues. MWA, HIFU, and SBRT are all relatively nascent technologies, though preliminary data regarding their use is promising, with overall survivals similar to RFA. These technologies expand upon the patients eligible for local ablation by offering different technical advantages as compared to RFA.

Tandem approaches with combination of local ablation such as RFA in addition to locoregional therapies such as TACE have already been explored with promising results demonstrating improved overall survival as compared to RFA alone. In addition, new technologies such as IRE are currently being clinically investigated to further expand the patients eligible to safely undergo local ablation.
